# Fetal-Derived Immune Cells at the Roots of Lifelong Pathophysiology

**DOI:** 10.3389/fcell.2021.648313

**Published:** 2021-02-23

**Authors:** Elvira Mass, Rebecca Gentek

**Affiliations:** ^1^Developmental Biology of the Immune System, Life & Medical Sciences (LIMES) Institute, University of Bonn, Bonn, Germany; ^2^Centre for Inflammation Research & Centre for Reproductive Health, The Queen’s Medical Research Institute, University of Edinburgh, Edinburgh, United Kingdom

**Keywords:** erythro-myeloid progenitors, macrophages, mast cells, developmental origins of health and disease, layered hematopoiesis, cancer, neurological disease, atopic disease

## Abstract

Tissue-resident innate immune cells exert a wide range of functions in both adult homeostasis and pathology. Our understanding of when and how these cellular networks are established has dramatically changed with the recognition that many lineages originate at least in part from fetal sources and self-maintain independently from hematopoietic stem cells. Indeed, fetal-derived immune cells are found in most organs and serous cavities of our body, where they reside throughout the entire lifespan. At the same time, there is a growing appreciation that pathologies manifesting in adulthood may be caused by adverse early life events, a concept known as “developmental origins of health and disease” (DOHaD). Yet, whether fetal-derived immune cells are mechanistically involved in DOHaD remains elusive. In this review, we summarize our knowledge of fetal hematopoiesis and its contribution to adult immune compartments, which results in a “layered immune system.” Based on their ontogeny, we argue that fetal-derived immune cells are prime transmitters of long-term consequences of prenatal adversities. In addition to increasing disease susceptibility, these may also directly cause inflammatory, degenerative, and metabolic disorders. We explore this notion for cells generated from erythro-myeloid progenitors (EMP) produced in the extra-embryonic yolk sac. Focusing on macrophages and mast cells, we present emerging evidence implicating them in lifelong disease by either somatic mutations or developmental programming events resulting from maternal and early environmental perturbations.

## Introduction

It is now widely recognized that many non-communicable diseases have developmental origins, brought about by somatic mutations or environmental perturbations during gestation and in early life. Immune dysregulation is a common denominator in the etiology of these diseases, and can even directly cause pathology. Indeed, immune cells have many functions beyond protective immunity, for example in controlling tissue homeostasis. The first immune cells seed developing tissues during organogenesis, and unlike previously thought, appear fully functional already at these early stages. Moreover, we have recently come to realize that fetal-derived cells persist and self-maintain in adult tissues. This is true for macrophages and mast cells derived from erythro-myeloid progenitors (EMP) generated in the yolk sac (YS) before the emergence of hematopoietic stem cells (HSC). Their ontogeny and proliferative capacity make EMP-derived cells particularly vulnerable to early life perturbations and identify them as potential transmitters of long-term effects on health and disease.

Here, we explore this notion, focusing on EMP and their cellular progeny. Because they allow establishing *in vivo* lineage and cause-consequence relationships between perturbation of immune development and pathology, we will primarily discuss experimental animal studies. However, where possible, we will also discuss relevant human data, especially those that benefitted from recent technological advancements such as single-cell RNA-sequencing. We will briefly summarize our current understanding of fetal hematopoiesis and its contribution to adult tissue-resident immune compartments/landscapes. Having established their normal developmental trajectories, we will then discuss a growing body of literature supporting the notion that mutations affecting EMP or exposure to adverse early life environments render macrophages and mast cells pathogenic in conditions as diverse as neurological or atopic disease and cancer.

### Layered Hematopoiesis

Traditionally, HSC found in the bone marrow (BM) have been regarded as the sole, lifelong source of all immune cells. This view has changed with the recognition that many lineages originate at least in part from fetal sources and self-maintain independently from HSC. Indeed, we now appreciate that fetal-derived cells comprise varying proportions of the resident immune compartments in most adult organs and serous cavities.

**FIGURE 1:**
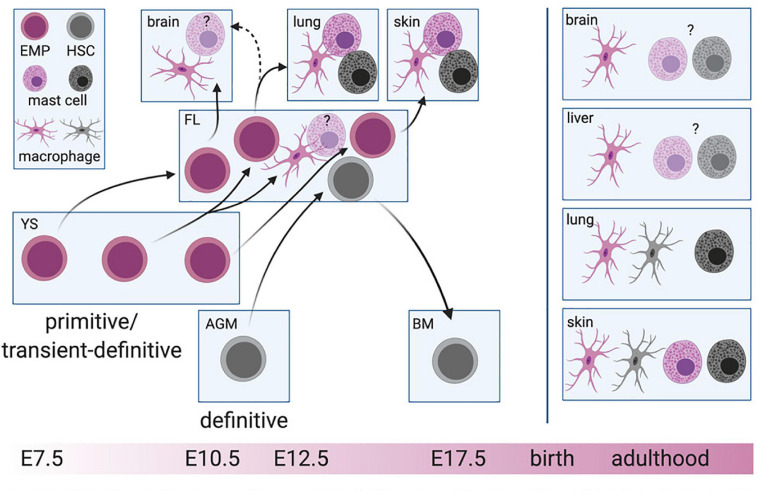
Layered hematopoiesis. The primitive and transient-definitive waves in the yolk sac (YS) give rise to erythro-myeloid progenitors (EMP) that will colonize the fetal liver (FL) consecutively. EMP arising at early stages will differentiate into macrophages and probably mast cells in tissues developing early during embryogenesis (brain, liver), while EMPs at later stages remain in the FL and will give rise to the same cell types driven by the demand of later developing tissues, such as the lung and skin. Hematopoietic stem cells (HSC) develop in the aorta-gonad-mesonephros (AGM) region before colonizing the FL. After the formation of the bone marrow (BM), HSC migrate to the BM cavity where they will constantly give rise to short-lived macrophages and mast cells. The cell of origin for mast cells and macrophages is color-indicated. For simplicity, blood-circulating intermediate precursor stages between EMP and macrophages are not depicted. Created with BioRender.com.

The production of hematopoietic progenitors is initiated early during mammalian development. Although low-grade hemogenic capacity might also exist in the BM during a brief perinatal window (Yvernogeau et al., 2019), *de novo* hematopoiesis is otherwise restricted to fetal stages. Fetal hematopoiesis occurs in several waves that differ in time and space but partially overlap (Figure 1). These distinct waves also differ in their lineage output, as we will discuss in more detail below.

#### Hematopoietic Waves

The first hematopoietic progenitors are produced in the extra-embryonic YS. In the mouse, this occurs on day 7 of embryonic development (E7.0) (Wong et al., 1986). This so-called **primitive** wave generates erythrocytes, megakaryocytes (Tober et al., 2007), and possibly also the first macrophages that colonize the embryo proper, at least microglia in the brain parenchyma (Alliot et al., 1999; Ginhoux et al., 2010). Primitive hematopoiesis is followed by the production of **erythro-myeloid progenitors (EMP)**, a second wave sometimes called transient or **transient-definitive** (Moore and Metcalf, 1970; Palis et al., 1999; Tober et al., 2007). EMP are generated between E8.5 and E10.5 from hemogenic endothelial cells of the YS (Gomez Perdiguero et al., 2015; McGrath et al., 2015). In addition to their erythrocyte, granulocyte, and megakaryocyte potential, EMP readily differentiate into macrophages, monocytes, granulocytes, and mast cells *in vivo* (Gomez Perdiguero et al., 2015; Hoeffel et al., 2015; Mass et al., 2016; Gentek et al., 2018a; Li et al., 2018). Finally, definitive HSC emerge in a third wave that originates from the main arteries of the embryo proper, particularly the aorta-gonad-mesonephros (AGM) region (Boisset et al., 2010), although hemogenic activity has been reported at additional sites including the umbilical and vitelline arteries, placenta, heart and the hindbrain-branchial arch region of the head (Alvarez-Silva et al., 2003; Gekas et al., 2005; Zeigler et al., 2006; Li et al., 2012, 2016; Gordon-Keylock et al., 2013; Solano et al., 2014; Shigeta et al., 2019). Like EMP, HSC derive from hemogenic endothelial cells (De Bruijn et al., 2002; Boisset et al., 2010). They transiently colonize the fetal liver to expand and ultimately settle in the BM, where they persist lifelong. HSC are operationally defined by their potential for self-renewal and multi-lineage reconstitution upon transplantation into irradiated adult hosts. Based on these criteria, additional populations of HSC have been described (Beaudin et al., 2016). However, their contribution to adult BM HSC remains controversial. Resolving this requires appropriate models that do not rely on transplantation but instead, allow assessment of persistence and lineage output under physiological conditions *in vivo*., e.g., using the Polylox barcoding model (Pei et al., 2017). Therefore, we propose to refer to these progenitors as fetal-restricted or **transient HSC** and distinguish them from **adult-type HSC** found in the BM.

This complex process generates what has been termed a “**layered immune system,**” in which certain lineages have distinct origins throughout life, whereas others remain entirely or in part of fetal origin. While originally postulated for innate-like B1 and conventional B(2) cells (Herzenberg and Herzenberg, 1989; Herzenberg, 2015), this concept now appears to be more globally applicable, in particular to long-lived tissue-resident innate cells, including macrophages, mast cells, and innate lymphocytes (Gentek et al., 2018b; Schneider et al., 2019; Dege et al., 2020; Simic et al., 2020).

#### Human Hematopoiesis

Similarly to mice, also human hematopoietic waves appear sequentially at distinct sites, with EMP-like cells in the YS that produce erythrocytes, as well as macrophages, megakaryocytes (reviewed in Julien et al., 2016) and mast cells (Popescu et al., 2019; Bian et al., 2020). Once the blood circulation is established, EMP colonize the fetal liver where they are joined by definitive HSC coming from the AGM (Julien et al., 2016). Recent single-cell RNA-sequencing studies of human fetal tissue provide further evidence for the evolutionary conserved hematopoietic development and molecular programs of individual cell types (Popescu et al., 2019; Bian et al., 2020; Cao et al., 2020), supporting the notion that also in humans EMP generate long-lived immune cells that are self-maintained independently of HSC. At least for tissue-resident macrophages, this is now an accepted concept, since transplant patients harbor donor macrophages in transplanted organs such as the lungs (Eguíluz-Gracia et al., 2016; Nayak et al., 2016), skin (Kanitakis et al., 2011), and heart (Bajpai et al., 2018) for many years. Taken together, evidence is mounting that adult human tissues harbor fetal-derived cells, therefore, supporting the use of the mouse as a powerful model system for studying human immune cell development and function.

#### Distinct Ontogeny = Distinct Function?

Layered hematopoiesis has fundamental implications: Developmentally distinct immune cells might exert discrete, non-overlapping functions, at least within defined stages of development or non-homeostatic conditions, as we have previously discussed (Geissmann and Mass, 2015; Schultze et al., 2019). This is true for pancreatic and lung cancer, in which macrophages derived from fetal progenitors and monocytes have distinct tumor-promoting and anti-tumor roles (Zhu et al., 2017; Loyher et al., 2018). Particularly the continuous development of new tools to efficiently target macrophages of distinct origins will pave the way to understanding complementarity functions of developmentally distinct macrophages. This has been shown in a stroke model, where activation and proliferation of EMP-derived microglia rely on a transient influx of monocyte-derived macrophages, which thereby conjointly control the regeneration of neuronal tissue (Werner et al., 2020).

Their developmental pattern also renders layered lineages particularly vulnerable to early genetic and environmental perturbations, which might translate into long-lasting or even permanent effects on later-life health and disease. While this might also apply to transient and adult-type HSC, as recently discussed (Apostol et al., 2020), we here focus on HSC-independent hematopoiesis, i.e., EMP and their effector cell progeny, because their developmental trajectories are increasingly well understood in health, and evidence is mounting that disruption of their normal blueprint mediates adult disease.

### EMP–From Transient Fetal Hematopoiesis to Lifelong Immune Landscapes

Although this three-wave model is widely accepted and experimental data are usually interpreted within the framework of its nomenclature, it likely represents an oversimplification of fetal hematopoiesis: Different progenitors share expression of common surface markers (e.g., CD45, Csf1r, CD41, Kit, CD16/32) and their production partially overlaps in space and time. Therefore, at present, no single fate-mapping model can unequivocally pinpoint distinct pre-HSC waves with precision, and conclusions on the origins of immune cells should be drawn with caution and from several *in vivo* models in a complementary manner. Negligence to do so-i.e., (over)interpretation of *in vivo* data from single fate-mapping models, intermingling with *in vitro* assays that do not necessarily reflect *in vivo* lineage output, and not accounting for developmental events during embryogenesis-has stirred an ongoing debate about the exact nature and identity of hematopoietic progenitors, their emergence and their contribution to distinct cell types (Perdiguero et al., 2015; Sheng et al., 2015; Ginhoux and Guilliams, 2016; Perdiguero and Geissmann, 2016; Palis, 2017).

One lingering controversy is the question whether EMP also generate lymphocytes. It has long been appreciated from *ex vivo* and progenitor transplantation assays that lymphoid potential precedes the onset of adult-type HSC-dependent hematopoiesis and that certain innate(-like) lymphocytes even remain of fetal origin throughout life (Hayakawa et al., 1985; Liu and Auerbach, 1991; Payer et al., 1991; Huang et al., 1994; Godin et al., 1995; Yokota et al., 2006; Yoshimoto et al., 2011; Lin et al., 2014). A series of recent fate-mapping studies have corroborated this notion *in vivo* with the demonstration that several lymphoid lineages such as dendritic epidermal T cells (DETC), lymphoid tissue inducer (LTi), and natural killer (NK) cells first emerge independently of adult-type HSC (Gentek et al., 2018b; Dege et al., 2020; Simic et al., 2020).

In trying to further pinpoint the exact sources of the first lymphocytes, the concept of YS-derived lympho-myeloid-restricted progenitors (LMP) has been put forward (Böiers et al., 2013), and LMP have been proposed to be the first progenitors seeding the developing thymus (Luis et al., 2016). However, the LMP denotation is in part based on co-expression of genes associates with both, myeloid and lymphoid lineages (Böiers et al., 2013; Zhu et al., 2020), which does not necessarily equal differentiation into these lineages. Indeed, this notion has recently been challenged with the demonstration that YS progenitors, despite transient expression of lymphoid-associated transcripts (*Il7r*, *Rag2*, *Rag1*), do not generate lymphocytes *in vivo* (Elsaid et al., 2020).

The LMP controversy illustrates how difficult it often remains to assign the origin of fetal-derived immune cells to specific progenitors, even with genetic fate mapping. The models used often rely solely on the temporal distinction of waves, the resolution of which is insufficient for progenitors produced between E8.5 and E10.5. Whilst DETC and fetal LTi are likely not EMP-derived based on the absence of labeling in several models (Gentek et al., 2018b; Elsaid et al., 2020; Simic et al., 2020), it has recently been suggested that the first fetal NK cells originate at least in part from EMP (Dege et al., 2020). However, this was assessed in a single model with relatively low labeling induced at time points for which the contribution of other progenitors cannot be excluded (Dege et al., 2020). Thus, the true physiological contribution of EMP to the first lymphocytes remains to be determined *in vivo* using additional, complementary approaches.

#### When Is an EMP and EMP?

Possibly the most heavily debated controversy concerns the precise origins of fetal-derived immune cells found in adult organs, in particular tissue-resident macrophages. While many scientists might dismiss this as a pure developmental biologists’ or even semantics problem, we believe it is essential to precisely dissect the ontogeny of those immune cells that remain lifelong within tissues, where they undergo continuous genetic and epigenetic changes that may eventually influence or perturb organ homeostasis. In an attempt to delineate different progenitor waves, the nomenclature of “early” and “late” EMP (or EMP1 and EMP2) has been introduced, which proposes that early EMP (produced at E7.5) belong to the primitive wave, while late EMP (starting at E8.5) express the transcription factor c-Myb and thus originate from the second, definitive wave of hematopoiesis (Hoeffel et al., 2015; Ginhoux and Guilliams, 2016). Because of these discrepancies in the definition of pre-HSC waves and the tools used to target them, different groups consider e.g., microglia, the brain-resident macrophages, derived either purely from the primitive (Alliot et al., 1999; Ginhoux et al., 2010; Hoeffel et al., 2015) and/or from the second wave (Kierdorf et al., 2013; Gomez Perdiguero et al., 2015; De et al., 2018). The concept of early and late EMP has also been applied to mast cells, and it has been speculated that their mast cell progeny differs in their longevity (Li et al., 2018). However, this has not been experimentally addressed, and could simply reflect responses to environmental differences encountered by progenitors recruited at distinct time points.

Indeed, it stands to reason that the strict segregation of early and late EMP based on timing (i.e., the time point of label induction in inducible fate-mapping models) and c-Myb expression is rather artificial and does not reflect the real-life scenario. Rather, early and late EMP might represent two extremes of the same wave, “captured” by different labeling approaches. We propose that EMP represent a continuous product of the YS hemogenic endothelium generated between E7.5 and E10.5, which intrinsically have the same differentiation potential, irrespective of their time of emergence. This is supported by clonogenic assays for EMP obtained from the YS at these different time points and fetal liver EMP at E12.5 (Gomez Perdiguero et al., 2015; Dege et al., 2020). *In vivo*, EMP heterogeneity and their actual lineage output would be dictated by stage-specific signals co-opting cell-autonomous transcriptional networks to meet the current demands for immune cells. Such a demand-driven model could easily be reconciled with existing experimental data: Macrophages are considered integral to organogenesis and thus, need to colonize tissues as they develop. At E7.5, YS EMP therefore primarily give rise to microglia, while at E10.5, they mainly produce macrophages for tissues that develop later during embryogenesis, e.g., the lung (Figure 1). Although their functions during development remain largely enigmatic, mast cells might contribute to fine-tuning of the nerve and vasculature networks, at least in the cornea (Liu et al., 2015). Rather than at the onset of organ development, they might thus be required only at later stages, in line with EMP starting to produce mast cell-committed progenitors at E12.5.

To respond to these demands, EMP need to produce committed progenitors that can invade tissues via the circulation. This can occur via different routes, some of which involve migration to the fetal liver (Stremmel et al., 2018), where they provide a hematopoietic reservoir at least until E16.5. The subsequent dynamics of EMP in the fetal liver and other hematopoietic organs, as well as their various differentiation trajectories that contribute to the layered immune system in each organ remain largely elusive, but their reconstruction will be feasible with the advent of single-cell technologies in combination with novel fate-mapping models.

### Developmental Programming of EMP-Derived Cells by Adverse Early Life Environments

It is now firmly established that the likelihood of developing a non-communicable disease in adulthood is strongly influenced by environmental factors in early life, including the fetal period (Barker, 2004). This concept is known as DOHaD and is deeply rooted in epidemiological studies, which have since been backed up by experimental data. Pioneering work focused on the consequences of maternal malnutrition on offspring health and found correlations with obesity, cardiovascular disease, hypertension, and diabetes (Roseboom et al., 2001; Barker et al., 2009; Ravelli et al., 2010). However, similar phenomena have since been observed for a wide range of adversities and (often chronic) pathologies, ranging from other dietary and lifestyle factors (e.g., maternal obesity, smoking), maternal disease and infection (such as maternal allergy), exposure to environmental pollutants (e.g., diesel exhaust, endocrine-disrupting chemicals) and psycho-social stress, which collectively increase the susceptibility to chronic inflammatory, atopic, auto-immune and neurological disease as well as cancer. A dysregulated immune response is common to all these pathologies.

Whilst similar considerations apply to environmental perturbations in the perinatal period, which is arguably important for shaping the immune system in response to microbial exposures and colonization, fetal development normally occurs in a tightly controlled intra-uterine environment and thus, represents a critical window of vulnerability. As outlined above, the fetal period also overlaps with key events of immune development, strongly suggesting that environmental insults experienced *in utero* impact later-life health and disease by programming offspring immunity.

#### EMP-Derived Cells as Mediators of DOHaD

**FIGURE 2:**
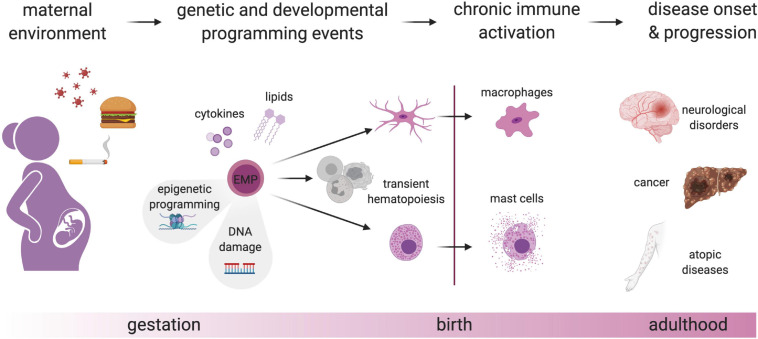
Genetic and developmental programming of fetal-derived immune cells. Perturbation of the maternal environment e.g., by nutrition, smoking, and infection, leads to the production of mediators such as cytokines and lipids that will lead to epigenetic alterations in EMP and thereby, chronic activation of EMP-derived immune cells after birth. This switch from a homeostatic to a pro-inflammatory state due to developmental programming may cause or contribute to different diseases. In addition to naturally occurring somatic mutations in proliferating EMP and their progeny, maternal-derived hazardous materials may lead to DNA damage, which could eventually lead to a change in transcriptional programs and chronic activation of tissue-resident immune cells. Created with BioRender.com.

At least three requirements must be met for environmental perturbations during fetal development to have long-lasting effects that can persist into adulthood: Signals must (1) be conveyed to the developing fetus, either directly by crossing the placental barrier, or by eliciting an inflammatory response at the fetal-maternal interface. Such signals must (2) be sensed by physiological systems equipped to respond to environmental stimuli, and (3) these systems must persist and undergo long-lasting imprinting or programming. This applies to the immune system (Palmer, 2012; Marques et al., 2013; Balistreri et al., 2019), and in particular to long-lived fetal-derived cells such as macrophages and mast cells, making them prime candidate mediators of long-term adverse effects (Figure 2).

Programming of these immune cells might act on different levels and manifest in several ways that are non-mutually exclusive. Adverse environments could directly act on individual cells, modulating their effector functions, causing e.g., a long-lasting shift in the balance between type I and II immunity (Veru et al., 2014). Adverse conditions during development could also affect the cellular longevity and self-maintenance capacity of individual cells, thereby ultimately modifying the composition or cellular dynamics of adult immune cell populations, which might result e.g., in higher-than-normal dependence on recruitment of BM progenitors. Functional perturbations might also result from impaired or altered crosstalk of programmed immune cells with each other and the non-hematopoietic stroma within their niches (Hsu et al., 2014; Guilliams and Scott, 2017; Naik et al., 2018; Zhou et al., 2018; Chakarov et al., 2019). Finally, in addition to local effects within their tissues of residence, developmentally programmed immune cells might also impact pathologies systemically or at other sites, for example, via long-range inter-organ trafficking, a phenomenon appreciated in recent years for infectious and inflammatory conditions (Huang et al., 2018; Leyva-Castillo et al., 2019). Thus, programming of fetal EMP-derived immune cells might result in increased susceptibility to a range of pathologies, examples of which we will discuss in the following.

#### Neurological Disorders

The DOHaD have been described half a century ago, directly linking prenatal nutrition to mental disorders (Zamenhof et al., 1966; Weinberger, 1987). Before the fetal origins hypothesis, the fetus was rather seen as a “perfect parasite”-absorbing what it needed but protected from nutritional damage inflicted on the mother (Susser and Stein, 2009). A major change in society was achieved in 1968 after results of a conference on early brain development and cognition were featured in newspapers stating that early nutritional deprivation causes irreparable damage to the brain and consequently to cognition (Scrimshaw et al., 1968). Since then, scientists aimed at understanding the mechanisms that can influence the developmental programming of the brain using animal models. By now, the research focus has switched from studying maternal undernutrition (e.g., human subjects born during the Dutch hunger winter) to maternal overnutrition, with nowadays ∼39% of women being overweight or obese, as well as pollution and maternal immune activation (MIA) models, with poly(I:C) or lipopolysaccharide (LPS) representing viral and bacterial infection, respectively. Intriguingly, maternal obesity in humans has been associated with cognitive impairment, learning disability, anxiety, and attention deficit hyperactivity disorder (Hatanaka et al., 2017). Similarly, in rodent models maternal obesity leads to marked changes in anxiety, learning behavior and memory in the offspring (Cordner and Tamashiro, 2015).

In light of the regulation of gene expression by metabolites and MIA, epigenetic regulation of gene expression is currently the proposed mechanism of adverse intergenerational effects (Nardone and Elliott, 2016; Contu and Hawkes, 2017). However, the identity of the cell types undergoing adaptations of epigenetic marks upon maternal obesity and their long-term functional consequences for brain development and function are not fully defined.

In the healthy developing brain, microglia interact with neuronal progenitors and regulate their cell numbers (Cunningham et al., 2013). They are key modulators of angiogenesis (Fantin et al., 2010), contribute to myelinogenesis (Wlodarczyk et al., 2017) and oligodendrogenesis (Shigemoto-Mogami et al., 2014), and control synaptic development and plasticity (Paolicelli et al., 2011; Schafer et al., 2012; Parkhurst et al., 2013; Miyamoto et al., 2016), and brain connectivity (Squarzoni et al., 2014). Microglia regulate brain development and function primarily through phagocytosis and paracrine signaling. Synapse elimination, also called synaptic pruning, is needed to remove the excess of synapses that has been established in the early postnatal stage. Synaptic pruning depends on complement proteins that tag inappropriate synaptic connections for phagocytosis by microglia. Dysregulation of the complement cascade leading to under- or over-pruning has been implicated in neurodevelopmental disorders (Presumey et al., 2017).

In the adult brain, microglia actively contribute to learning and memory by surveying and modulating multiple synaptic structures (Tremblay et al., 2010; Wang et al., 2020), controlling neuronal activity (Badimon et al., 2020), and clearing apoptotic neural progenitor cells in the dentate gyrus (Sierra et al., 2010). In contrast, excessive activation of microglia leads to changes in synaptic transmission (Pascual et al., 2012), enhanced phagocytotic activity, and can contribute to or even cause neurodegenerative diseases (Mass et al., 2017; Song and Colonna, 2018).

This overwhelming collection of the homeostatic functions of microglia, their longevity, and their ability to acquire epigenetic memory (Wendeln et al., 2018) makes them a prime candidate for the intergenerational transmission of persistent changes inflicted by the maternal environment causing neurodevelopmental and neurodegenerative pathologies. Past difficulties in proving this cause-consequence relationship on a transcriptional level may be due to the transient nature of perturbations in microglia development (Matcovitch-Natan et al., 2016), which nevertheless might have far-reaching implications for brain function across the lifespan, during which the body keeps accumulating adverse environmental challenges.

#### Allergy and Atopic Diseases

Atopic diseases are caused by IgE-mediated allergic reactions that represent exaggerated immune responses to otherwise harmless substances like pollen, dander, and certain types of food. Mast cells are the central mediators of these responses (Reber et al., 2012). Upon the first encounter of such allergens, IgE binds to its high-affinity receptor Fcer1 on mast cells, which get activated following subsequent allergen exposures, resulting in rapid release of a variety of very potent effector molecules such as histamine, serotonin, proteases, and lipid mediators. These cause symptoms of varying severity that range from sneezing, itch, wheeze, rashes, gastro-intestinal manifestations like vomiting and diarrhea to life-threatening anaphylaxis. Depending on the nature of the allergen and exposure route, atopic disease primarily affects the skin and airways, presenting as atopic dermatitis, allergic rhinitis, or asthma. Their overall prevalence has dramatically increased and continues to rise (Asher et al., 2006; Anandan et al., 2010; Pedersen et al., 2011; Backman et al., 2017).

It has previously been recognized that allergic sensitization might occur prenatally (Piccinni et al., 1993; Kihlström et al., 2003; Boyle and Tang, 2006), and a family history of atopy is a well-established risk factor (Tariq et al., 1998; Sugiyama et al., 2007; Indinnimeo et al., 2016). This correlation is consistently higher for maternal compared to paternal atopy (Aberg, 1993; Litonjua et al., 1998; Bracken et al., 2002; Johnson et al., 2002; Liu et al., 2003; Wu et al., 2012), further implicating the maternal microenvironment. A recent study in mice provided experimental evidence in support of this notion with the demonstration that fetal mast cells sensitized *in utero* by maternal IgE crossing the placental barrier mediate airway and skin inflammation upon postnatal re-exposure (Msallam et al., 2020). Given its potential to quickly amplify disease prevalence from one to the next generation, programming by maternal disease might have contributed to the dramatic surge in atopic disease observed within just a few decades.

Other early life risk factors implicated in atopic disease are air pollution (Hsu et al., 2015; Deng et al., 2016) and parental smoking (Martinez et al., 1992; Raherison et al., 2007), as well as maternal stress (Andersson et al., 2016; Chang et al., 2016; Magnus et al., 2018) and obesity (Reichman and Nepomnyaschy, 2008). For maternal obesity and smoking, the correlation appears to be stronger for offspring allergic asthma (Krämer et al., 2004; Raherison et al., 2007; Ekström et al., 2015), underscoring that differences may exist in the etiology and programming of distinct atopic disorders, which might be explained by locally restricted production of IgE (Coëffier et al., 2005; Takhar et al., 2007). The mechanisms by which these factors impact susceptibility to atopic disease and the cellular targets they are acting on are less well defined. However, mast cells are also involved in the more chronic stages of allergic inflammation (Galli and Tsai, 2012), a process that macrophages might also impact on (Zasłona et al., 2014). Of note, populations of fetal-derived macrophages exist in the skin (Kolter et al., 2019) and airways (Loyher et al., 2018; Liu et al., 2019). Exposure to a perturbed intra-uterine environment could thus render mast cells and macrophages pro-inflammatory by signals other than antigen-specific IgE, which might lower their threshold for activation or prevent them from returning to baseline. This could be mediated by an inflammatory response in the mothers, which represents a shared feature of the above-mentioned early life risk factors. As introduced, MIA and even more complex environmental perturbations like pollution and maternal obesity can be modeled *in vivo*. Combined with genetic tools, future work can thus determine if and how mast cells and macrophages are programmed into a chronic inflammatory state promoting atopic diseases. Although causality with atopic disease remains to be established, evidence is mounting that mast cells are indeed hyperactivated by stress experienced during development. This will be discussed in the following.

#### Irritable Bowel Syndrome

Early life stress is also a major risk factor for gastrointestinal diseases, including irritable bowel syndrome (IBS) (Talley et al., 1994; Bradford et al., 2012). IBS is a chronic disorder characterized by abdominal pain, diarrhea, bloating, and vomiting. Its etiology is incompletely understood, but deregulated mast cell activation is widely recognized as a key pathological event (Zhang et al., 2016; Boeckxstaens, 2018). In patients, the number of mast cells located in the proximity of enteric nerves is significantly increased, as is the frequency of their degranulation, features that correlate with the degree of abdominal pain (Park et al., 2003; Barbara et al., 2004). Furthermore, mast cell activation syndrome patients are often affected by similar gastrointestinal symptoms (Hsieh, 2018), and pharmacological targeting of mast cells using antagonists of receptors for histamine and serotonin or mast cell stabilizers has proven beneficial for some patients (Klooker et al., 2010; Zhang et al., 2016). Substantial evidence links early life stress, mast cell hyperactivation and susceptibility to IBS. In a porcine model, early weaning stress induces chronic diarrhea and intestinal permeability, indicative of IBS-like symptoms, which are accompanied by elevated numbers and baseline degranulation of intestinal mast cells are observed (Pohl et al., 2017).

Similarly, in mice, early life stress potentiates mast cell- glia and -neuron interactions in the myenteric plexus in a histamine-dependent manner (McClain et al., 2020). While mast cells normally interact with the enteric nervous system to regulate intestinal homeostasis, i.e., bowel movements, barrier defense and maintenance, inappropriate mast cell activation as observed following early life stress appears to sensitize enteric neurons and glia, resulting in chronic pain, altered gut permeability and motility (Barbara et al., 2007; McClain et al., 2020). Mechanistically, mast cell hyperactivation could be the consequence of epigenetic inactivation of CRF2 (corticotropin-releasing factor receptor subtype 2), which normally limits mast cell degranulation through inhibiting store-operated calcium signaling (Ayyadurai et al., 2017). Future studies should account for the particular vulnerability of (immune) development during the prenatal period and use genetic models to establish if hyperactivation of intestinal mast cells and potentiation of their neuronal interactions are causal.

#### Cancer

Macrophages and mast cells densely populate the stroma of most solid tumors, where they can have either tumor-promoting or -suppressive functions. At present, this complexity represents a major hurdle for the clinical exploitation of these cells to diagnostic, prognostic or therapeutic benefit. Their impact on tumor growth can be different within distinct cellular neighborhoods (Schürch et al., 2020) and thus, is at least in part determined by local microenvironmental signals. However, developmental characteristics might equally contribute. Sizeable populations of fetal-derived immune cells persist in most healthy adult tissues, and could thus be recruited to malignant lesions. Intriguingly, a recent study suggests that specific interactions between fetal-like macrophages and fetal-associated endothelial cells provide an immuno-suppressive environment promoting hepatocellular carcinoma (Sharma et al., 2020), a phenomenon that might be more globally true. Furthermore, fetal-derived macrophages originating from YS EMP appear to promote tumor progression and fibrosis in a murine model of pancreatic ductal adenocarcinoma, whereas monocyte-derived macrophages might be involved in anti-tumor immunity (Zhu et al., 2017). Of note, macrophages phenotypically resembling these developmentally distinct populations can also be identified in human tumors. Similarly, in murine lung carcinoma, fetal-derived interstitial macrophages co-exist with monocyte-derived ones, and promote tumor growth and spread, respectively (Loyher et al., 2018).

In addition to their ontogeny, environmental challenges experienced during development might impact the functions of tumor-associated macrophages and mast cells. Epidemiologically, the risk of developing cancer has been associated with “stressful” adverse childhood experiences, such as trauma, maltreatment, or abuse, albeit inconsistently (Felitti et al., 1998; Fuller-Thomson and Brennenstuhl, 2009; Brown et al., 2010). This is in part attributable to the retrospective nature of these studies and self-reporting of stressful experiences. However, a prospective birth cohort study found a two-fold increased risk for cancer in individuals who experienced more than 2 stressful events in childhood, even when confounding factors were accounted for Kelly-Irving et al. (2013). In line with these findings, a recent study reported a higher melanoma burden in mice prenatally exposed to maternal stress (Hong et al., 2020). Whilst attributed to impaired T cell immunity, T cell recruitment, and activation are regulated by myeloid cells. EMP-derived mast cells persist in the adult (Gentek et al., 2018a; Li et al., 2018), and mast cells show signs of hyperactivation in *in vivo* models of maternal stress, as discussed above. Moreover, patients suffering from mast cell activation syndrome are also at a higher risk of developing solid tumors, including melanoma (Molderings et al., 2017). Programming of fetal-derived mast cells e.g., by prenatal stress might thus contribute to an overall melanoma-promoting environment.

### Genetic Perturbations in EMP and Their Progeny

#### Histiocytosis

Histiocytoses are rare diseases characterized by aberrant expansion of histiocytes, which is a historical term describing macrophages or dendritic cells. Langerhans cell histiocytosis (LCH) is one example, which presents a remarkable diversity of phenotypes ranging from subtle skin lesions and mild neurological symptoms to life-threatening disseminated disease. Since the discovery of recurrent somatic mutations in the MAPK signaling pathway, particularly BRAF^*V*600E^ (Badalian-Very et al., 2010; Satoh et al., 2012; Berres et al., 2014; Haroche et al., 2015; Diamond et al., 2016), known to be oncogenic in several human cancers (Davies et al., 2002), histiocytoses are now considered inflammatory myeloid neoplasms. Much like normal tissue-resident macrophages until recently, the mutant histiocytes were also thought to derive predominantly from BM progenitors. Yet, diverse phenotypes, particularly neurodegenerative and behavioral deficits that were retrospectively diagnosed in many patients (Cohen-Aubart et al., 2018; Héritier et al., 2018) as well as spontaneous regression of pediatric histiocytoses, are difficult to reconcile with mutated cells infiltrating from the BM, and hence, their causes remained a matter a debate.

Once the YS origin of adult microglia in mice was established (Ginhoux et al., 2010) and genetic models were available to target microglial progenitors, we introduced the BRAF^*V*600E^ mutation into the EMP lineage (Mass et al., 2017) to address the possibility that the clinical outcome of histiocytosis is not dictated by mutation of distinct progenitors in the adult BM (Berres et al., 2014, 2015), but rather by affecting one of the fetal hematopoietic waves. Indeed, all mice born with mutated microglia (∼15% BRAF^*V*600E+^) developed behavioral deficits and displayed chronically activated microglia, which eventually resulted in paralysis and neurodegeneration. In contrast, BRAF^*V*600E^ expression in all CD11c^+^ cells, i.e., dendritic cells and some tissue-resident macrophages, resulted in myeloid-cell tumors in the lung and spleen while targeting the whole hematopoietic system caused a leukemic phenotype and prenatal lethality (Mass et al., 2017). Due to the intentionally low targeting efficiency of EMP using the *Csf1r*^*MeriCreMer*^ model, we did not observe a large population of BRAF-mutated macrophages in other tissues. However, higher tamoxifen doses or different fate-mapping models may allow characterization of other organs, e.g., the bone or liver, where EMP-derived macrophages may play a causative role in the development of histiocytic lesions.

#### Mastocytosis

Mastocytosis is an umbrella term for a heterogeneous group of rare disorders characterized by aberrant clonal expansion of mast cells, for which curative treatments are not available. These disorders are classified as myeloproliferative neoplasms and are often benign, but can also become malignant or associated with additional hematological malignancies (Valent et al., 2017). Symptoms caused by the release of bioactive mast cell mediators include skin reactions such as itching, gastrointestinal complications like nausea and diarrhea, as well as bone, joint and muscle pain, fatigue, and an increased risk of anaphylaxis.

Different forms of mastocytosis are distinguished according to the affected sites, disease onset, and clinical course. Reflecting the high abundance of dermal mast cells in health, mastocytosis usually involves the skin. In cutaneous mastocytosis, mast cell expansion is restricted to the skin, whereas systemic forms also manifest at additional sites such as the BM and internal organs like the liver, spleen, lymph nodes, and the gastrointestinal tract. Pediatric disease is predominantly of the cutaneous type, generally follows a milder course and usually regresses by adolescence. Adult onset mastocytosis, on the other hand, is frequently systemic and does not normally regress. The majority of adult patients present with stable, indolent disease and symptoms ranging from mild to moderate, however, adult mastocytosis can also progress into aggressive systemic disease or mast cell leukemia (Georgin-Lavialle et al., 2013; Berezowska et al., 2014), both of which have more severe symptoms and dismal prognosis.

Despite substantial progress in understanding the etiology of mastocytosis on a cellular and molecular level, the biological differences underlying the remarkably distinct clinical features of pediatric and adult mastocytosis remain enigmatic. Mastocytosis is caused by mutations in *KIT*, the tyrosine kinase receptor for stem cell factor, on which mast cell development and maintenance depends. With very few exceptions (Beghini et al., 2001; Akin et al., 2004; Tang et al., 2004; Hartmann et al., 2005; Zhang et al., 2006; Wasag et al., 2011; Speight et al., 2013), these mutations are somatic and activating in nature, resulting in constitutive or ligand-independent KIT signaling, enabling deregulated mast cell expansion. Such *KIT* mutations have been identified in aberrant mast cells of virtually all adult- and the majority of pediatric-onset mastocytosis patients (Frieri and Quershi, 2013). The most common mutation is a substituting point mutation in codon 186 (D186V), which has been reported in about 80% of adult patients. Although this particular mutation is less abundant in pediatric patients, it is still found in almost 40% of cases, and of the remainder, another approximately 40% carry activating mutations in other regions of the *Kit* gene (Bodemer et al., 2010; Frieri and Quershi, 2013). Auto-activating Kit mutations are thus a shared feature of pediatric and adult mastocytosis. While different types of mutations may be of prognostic relevance, they unlikely account for the striking clinical differences between the transient pediatric and chronic adult forms. Rather, different cells of origin (i.e., EMP- vs. HSC-derived) might be underlying the distinct clinical entities, paralleling the considerations for histiocytosis.

Intriguingly, spontaneous regression of pediatric and persistence of adult-onset mastocytosis bear close resemblance to the kinetics of normal mast cell development. In mice, YS EMP-derived mast cells are gradually diluted and, in most tissues, ultimately largely replaced by adult-type HSC-derived ones (Gentek et al., 2018a; Li et al., 2018). This change occurs in the first weeks of life, corresponding to puberty, which also marks the age at which pediatric (usually < 15 years) and adult-onset (>15 years) are clinically distinguished, as well as the time regression is observed in pediatric-onset patients. Thus, it is tempting to speculate that pediatric and adult mastocytosis have different cells of origin, namely YS-derived EMP and HSC. In support of this notion, activating *KIT* mutations have been identified in HSC and more committed downstream progenitors in the BM and peripheral blood of adult patients with systemic disease (Afonja et al., 1998; Jara-Acevedo et al., 2015; Jawhar et al., 2015; Grootens et al., 2019), but not pediatric patients. While this hypothesis awaits experimental confirmation, such a scenario would be reminiscent of activating *BRAF* mutations that have different pathological consequences depending on the progenitor they are affecting.

### Immune Cell Mosaicism in Cancer

Malignant transformation is a multi-hit process that requires cooperation between mutations in oncogenes and tumor suppressor genes within one cell. However, in addition to these cell-autonomous events, tumor growth further depends on interactions between malignant cells and their microenvironment (Hanahan and Weinberg, 2011). The so-called tumor stroma is densely populated by immune cells, which can have tumor-promoting or -suppressive functions that appear to be determined locally within their cellular neighborhoods (Schürch et al., 2020). Intriguingly, a recent study suggests that specific interactions between fetal-like macrophages and fetal-associated endothelial cells provide an immuno-suppressive environment promoting hepatocellular carcinoma (Sharma et al., 2020). However, although sizeable populations of fetal-derived immune cells persist in most healthy adult tissues, whether their mutation is causally involved in the emergence of solid tumors has not been addressed.

Here, we discuss a possible cooperative mechanism between tumor and EMP-derived cells that we term **intercellular complementation** and that would allow pre-malignant cells to evade immune surveillance. In genetics, complementation typically describes the combination of two genomes containing distinct recessive mutations that results in a mutant phenotype. In our case, neoplastic mutation of one cell type would be complemented by mutation of a neighboring tissue-resident immune cell, and only collectively would these mutations promote tumor growth. Such phenomena have been described in the fruit fly, where tumorigenesis is initiated by cooperating oncogenic mutations in Ras and Notch affecting neighboring epithelial cells (Brumby and Richardson, 2003), whilst mutations in Ras and genes affecting cell polarity cooperate to confer metastatic behavior (Pagliarini and Xu, 2003). The unrestricted growth of “winner” over “loser” epithelial cells is also termed cell competition, and applies not only to malignant settings, but also represents a well-known mechanism in developing tissues. Conversely, more recent work implicated cell competition in restraining clonal outgrowth of super-fit (pre-malignant) clones in tissues with high mutational burden (Bowling et al., 2019). This concept stems from deep sequencing work revealing a vast degree of mutational diversity in tissues from aged, healthy/non-diseased humans such as the skin (Martincorena et al., 2015), esophagus (Martincorena et al., 2018), and endometrium (Anglesio et al., 2017). Since every proliferating cell, including long-lived tissue-resident immune cells, accumulates a high number of somatic mutations throughout its lifetime, we hypothesize that immune cell mosaicism may play not only a contributing but causative role in cancer development and progression.

#### Intercellular Complementation of Mast Cells in Neurofibroma

Neurofibromatosis type 1 (NF-1) is a common genetic disorder caused by loss-of-function mutations in the *NF1* tumor suppressor gene, which encodes neurofibromin, a GTPase activating protein negatively regulating the activity of the proto-oncogene Ras. These mutations can arise spontaneously, though often are congenital. Patients frequently develop plexiform neurofibroma derived from Schwann cells, which are benign, but difficult to resect. Despite being a genetic disease, symptoms, clinical course, and severity are highly variable. This is at least in part explained by the fact that loss of heterozygosity (LOH) for *NF1* in Schwann cells alone is not sufficient to induce neurofibromas (Zhu et al., 2002). Strikingly, heterozygosity for Nf1 in mast cells elicits tumor formation in mice with biallelic loss of Nf1 in Schwann cells (Yang et al., 2008). Mechanistically, Nf1 heterozygosity appears to render mast cells more sensitive to Kit ligand, which attracts them to peripheral nerves and likely regulates their expansion and/or survival within the growing tumor. Consequently, pharmacological inhibition of Kit signaling inhibits tumor formation and attenuates tumor growth.

Although the tumor-promoting role for MC in neurofibroma has been demonstrated using BM transplantation into adult recipient mice, it is important to note that the onset for tumor development often is in childhood, and this is particularly true for plexiform neurofibroma (Ferner et al., 2007). It is therefore tempting to speculate that LOH in Schwann cells and mutations in EMP-derived mast cells complement one another to facilitate tumor growth. Since they are likely to experience less selection pressure than neoplastic Schwann cells, mast cells might be less prone to developing drug resistance and thus, represent the better therapeutic targets. It will be important to address if mutations in mast cells complement neoplastic cells also in other tumors with mast cell infiltrates, and whether this in part explains the conflicting results implicating them as either beneficial (Biswas et al., 2014; Siiskonen et al., 2015) or detrimental (Tóth-Jakatics et al., 2000; Ribatti et al., 2003a, b).

#### Intercellular Complementation of Tissue-Resident Macrophages

Similar to mast cells, also macrophages seem to participate in intercellular complementation. This is the case for example in a sporadic colorectal tumor model where reciprocal BM chimera studies indicate that tumor-associated macrophages (TAMs) with a constitutively active cytoplasmic hematopoietic cell kinase promote tumorigenesis (Poh et al., 2017). However, these macrophages derive from adult BM monocytes and are typically recruited to pre-existing malignant lesions, indicating that genetic mosaicism of TAMs is not the main driver of the disease. To our knowledge, there is so far no single study demonstrating the initial cellular interaction of fetal-derived tissue-resident macrophages and a pre-malignant cell resulting in tumor development. While following the early stages of malignant transformation may be experimentally challenging in mice, *Drosophila* is a powerful tool to manipulate different cell types genetically and in a mosaic fashion, and there are some parallels to mammals concerning macrophage development (Gold and Brückner, 2015). Here, a screen could be set up, e.g., by introducing Ras^*V*12^ or other oncogenes into hemocytes, the tissue-resident macrophages of the fruit fly, while using RNAi or overexpression in other cell types such as endothelial cells or neurons via mosaic analysis with a repressible cell marker (MARCM) to characterize the combination of two distinct genetic alterations that result in tumorigenesis.

In more general terms, the contribution of EMP-derived macrophages to the pre-metastatic niche can be studied in mice by genetically manipulating these cells using common fate-mapping drivers such as *Csf1r*^*MeriCreMer*^ or *Cx3cr1^*CreERT*^* models (Mass, 2018) in combination with metastatic cell lines. Since the liver is a common site for metastatic disease, Kupffer cells, as the resident macrophage population lining the hepatic sinusoids, are prime candidates to prevent or promote tumor metastasis. Kupffer cells are scavengers that phagocytose and eliminate circulating dead and dying cells, commensal bacteria, and other waste products that pass through the liver sinusoids. Thus, a homeostatic Kupffer cell is the first line of defense against incoming metastatic tumor cells (Bayon et al., 1996; Keirsse et al., 2018). In contrast, perturbance of its homeostatic function e.g., diminished phagocytic activity via depletion of Dectin-2 (Kimura et al., 2016) or persistent immune activation enhances cancer cell metastasis (Keirsse et al., 2018). In summary, it is becoming increasingly evident that EMP-derived macrophages are not just bystanders reacting to inflammatory events in their tissue of residence, but that they are active modulators of adult pathophysiology.

### Is Neurodegeneration a Kind of Cancer?

As described above, a BRAF^*V*600E^ mutation in the EMP lineage results in mutant microglia, thereby causing neurodegeneration (Mass et al., 2017). Similarly, another oncogene expressed in microglia-Ras^*V*12^ - is sufficient to activate microglia and lead to photoreceptor degeneration (Moriuchi et al., 2020). In contrast, microglial deletion of tumor suppressor genes such as Transforming growth factor-β activated kinase 1 (TAK1) (Goldmann et al., 2013) or p53 (Su et al., 2014; Aloi et al., 2015) is neuroprotective, suggesting that the underlying functional dichotomy of cancer genes in microglia may represent a mechanism that drives neurodegeneration in a non-cell-autonomous manner. It is therefore conceivable that other mutations accumulating during aging that are usually found in malignant cells will lead to chronic activation of microglia resulting in increased cell proliferation, cytokine expression, and phagocytosis. Since microglia are now considered to be a major genetic risk factor in many age-related neurodegenerative diseases such as Alzheimer’s and Parkinson’s disease (Bartels et al., 2020) it may well be that certain forms of these diseases are indeed due to “cancerous microglia”-a hypothesis that can likely be tested via deep sequencing of cell nuclei to detect mosaicism (Lee et al., 2018) in the near future. Encouragingly, albeit that the cellular target remains unknown in humans, there are ongoing phase 1–3 clinical trials using cancer kinase inhibitors to treat Alzheimer’s Disease (Fagiani et al., 2020).

### Open Questions and Future Directions

One overarching question remaining is why the animal kingdom relies on consecutive hematopoietic waves, with only the adult-type HSC remaining active later throughout life. We believe that evolution has selected for this layering to supply immune cells that meet the stage-specific demands of developing tissues.

#### Why Are Certain Immune Cells Needed Early?

At earlier stages, rather than providing protective immunity, these demands are likely homeostatic in nature. Indisputably, oxygen supply by YS-derived red blood cells is needed as soon as the heart starts beating, a time point at which the embryo proper is developmentally likely not equipped to give rise to HSC. This may be explained by the need for a state of physiological hypoxia in embryos, which is important for the proliferation and survival of hematopoietic precursors (Simon and Keith, 2008). Similarly, macrophages seem indispensable throughout early embryogenesis owing to their contribution to angiogenesis (Fantin et al., 2010), neurogenesis (Cunningham et al., 2013), BM formation (Jacome-Galarza et al., 2019), and many other developmental processes that we are just now beginning to understand. They are phylogenetically the oldest immune cell type, originally discovered by Ilja Metchnikoff in starfish larvae (Metchnikoff, 1905). Macrophage-like hemocytes are the only immune cells also in other invertebrates such as *Drosophila*, where they have prominent roles in development and immunity (Parsons and Foley, 2016), similar to tissue-resident macrophages in mice. For mast cells, the reasons for their production before the onset of HSC hematopoiesis are currently less clear. In both mice and humans, they colonize embryonic tissues with some delay compared to macrophages, indicating they might not contribute to the initial steps of organogenesis, but rather, organ maturation. Indeed, mast cells appear to regulate corneal nerve and vasculature as well as mammary gland branching (Lilla and Werb, 2010; Liu et al., 2015). Beyond our present focus on EMP-derived lineages, similar considerations also apply to other lineages with layered ontogeny, such as innate lymphocytes.

#### Protective Immunity and Immune Priming *in utero*?

In addition to developmental functions, fetal immune cells might protect from infections occurring during pregnancy or, following *in utero* priming, postnatal life. Infectious threats would arguably have to occur at frequencies high enough to impose a strong selection pressure for establishing immune cells this early. This might well be the case in nature and could explain why the developing fetus also invests energy in generating other, short-lived cell types such as granulocytes and NK cells, which will be replaced just a few days later.

At least longer-lived fetal immune cells might also undergo immune priming. Although it is the longstanding belief that the fetus is sterile, data is emerging that the human microbiome is seeded before birth and DNA of bacteria, fungi, and viruses has been detected in amniotic and meconium fluid (Stinson et al., 2019). These studies remain controversial due to contamination issues during sample acquirement and processing, but evidence is mounting that the fetal immune system can detect and respond to microbial compounds and other immune-stimulatory agents present at the fetal-maternal interface, regardless of their source. Sensitization of fetal mast cells by maternal IgE is in keeping with this (Msallam et al., 2020). While this promotes allergy postnatally, such intra-uterine immune priming does not necessarily have to be pathological, but could also be protective during later-life exposures.

#### Are Fetal-Derived Immune Cells Mediators of Lifelong Pathology?

Whatever their physiological functions, the presence of fetal-derived, proliferating immune cells in virtually all adult tissues makes the organism vulnerable to developmental programming events during gestation, as well as the accumulation of somatic mutations. These perturbations may shift their functions from homeostasis- to inflammation-promoting. Using mice as model organisms and applying environmental challenges such as MIA, maternal obesity, or smoking, we may be able to establish cause-consequence relationships between developmental programming of EMP-derived cells and pathophysiology in the offspring, which cannot be deduced from epidemiology. Combining such models with the ever-growing toolbox to target developmentally distinct immune cell populations or their hematopoietic progenitors will allow us to dissect their precise roles in adult disease onset and progression. Delineating the basic mechanisms shaping the functions of EMP-derived cells might ultimately inform if and how we could reverse their programming toward restoring homeostasis. Finally, such mechanistic studies in animals can now be complemented with deep sequencing efforts of single human cells, where somatic mutations in nuclear or mitochondrial DNA might serve as a readout for cellular origin, thus allowing us to study immune cell origin not only in patients, e.g., after transplantation, but also in healthy subjects.

## Author Contributions

RG and EM wrote the manuscript. Both authors contributed to the article and approved the submitted version.

## Conflict of Interest

The authors declare that the research was conducted in the absence of any commercial or financial relationships that could be construed as a potential conflict of interest.

## Abbreviations

AGM: aorta-gonad-mesonephros
BM: bone-marrow
DETC: dendritic epidermal T cells
EMP: erythro-myeloid progenitor
HSC: hematopoietic stem cell
IBS: irritable bowel syndrome
LTi: lymphoid tissue inducer
MIA: maternal immune activation
NK: natural killer
YS: yolk sac
